# When Distress Goes Unseen: Mental Health Recognition Failures in Language-Discordant Consultations

**DOI:** 10.3390/healthcare14182945

**Published:** 2026-09-10

**Authors:** Carmen Pena-Díaz, Fe Amalia García Santiago

**Affiliations:** 1 FITISPos Research Group, Department of Modern Languages, Universidad de Alcalá, 28801 Alcala de Henares, Spain; 2 INGEMM (Instituto de Genética Médica y Molecular)-Idipaz, Hospital Universitario La Paz, 28046 Madrid, Spain; feamalia.garcia@salud.madrid.org; 3 CIBERER (Centro de Investigación Biomédica en Red de Enfermedades Raras), Instituto Carlos III, 28046 Madrid, Spain

**Keywords:** psychological distress, early recognition, interpreter-mediated communication, multilingual healthcare, language access, migrant health, mental health integration, vulnerable populations

## Abstract

**Highlights:**

**What are the main findings?**

- In the analysed consultations, biomedical information was generally conveyed in recognisable form, while some explicit distress expressions and observable emotional cues were reduced, softened or not explored. These qualitative observations are illustrative and do not estimate comparative frequency.
- Observable responses to distress varied across the analysed non-mental-health encounters; the exploratory design does not permit attribution of this variation to departments, provider profiles, languages or interpreter characteristics.

**What are the implications of the main findings?**

- Effective language access should support the recognition of clinically relevant emotional distress as well as the accurate transfer of biomedical information.
- The findings suggest that multilingual hospital care may benefit from communication practices that preserve emotional meaning and from accessible procedures for further assessment when clinically indicated.

**Abstract:**

Background/Objectives: Early recognition of psychological distress is an important component of integrated healthcare, particularly among patients experiencing social and linguistic vulnerability. This hypothesis-generating study examined how observable expressions of distress were conveyed and addressed in multilingual hospital encounters. Methods: An exploratory qualitative analysis was conducted using fifteen interpreter-mediated consultations purposively selected from the Intercomsalud corpus because verified transcripts were available. The sample included consultations in Arabic (n = 6), Chinese (n = 2), English (n = 5), Russian (n = 1) and Ukrainian (n = 1). Coding distinguished (1) explicit verbal distress, (2) observable emotional cues, and (3) researcher-inferred distress; it also described full, partial or non-relay and the healthcare professional’s observable response within the recording. Results: In the analysed consultations, biomedical and procedural information was generally conveyed in recognisable form. Some explicit distress expressions and observable cues were reduced, reformulated or not explored, whereas other encounters showed sustained acknowledgement. The psychology consultation provided a suggestive, uncontrolled contrast and cannot establish a department effect. Conclusions: The cases illustrate that clinical-content relay and affective recognition can be examined as related dimensions, but the present sample primarily documents accurate clinical-content relay with variable affective recognition and does not empirically validate their full independence. Larger, independently coded studies with follow-up data are needed.

## 1. Introduction

Mental health integration into services that are not organised around mental health as their primary mandate has become a recognised priority, driven by the understanding that psychological distress frequently emerges—and may remain undetected—outside psychiatric and psychological care [1,2]. Paediatric wards, oncology units, obstetric services and primary care therefore function as potential sites of early recognition, whether or not their staff are specifically trained or equipped for this role. This is especially consequential for vulnerable populations, including children, older adults, people with disabilities and individuals experiencing social exclusion, whose distress may not present in ways that non-specialist professionals readily identify [3]. This broader responsibility is consistent with international policy frameworks that call for mental health promotion, prevention and care to be integrated across health systems rather than confined to specialist services [4]. It also reflects the principles of integrated, people-centred healthcare, in which services are expected to respond to individuals’ needs across the continuum of care and not solely to the disease or condition defining a particular consultation [5].

Migrant patients involved in language-discordant consultations constitute a further population at risk of recognition failure. Language barriers may restrict access to information, affect patient participation and increase the likelihood of clinically significant communication errors [6,7]. Existing research has also shown that interpreter-mediated encounters can reproduce power asymmetries: patients may be addressed in the third person, spoken about rather than spoken to and excluded from parts of the clinical decision-making process [8,9,10,11]. Research on language barriers and professional interpreting has demonstrated that access to trained interpreters can improve communication, comprehension and quality of care, but much of this evidence has evaluated success principally in terms of the accurate and complete transmission of clinical information [6,7].

This literature, including a separate analysis of the Intercomsalud corpus (Pena-Díaz, under review), has largely focused on epistemic authority: whose account of the clinical situation is treated as valid and how communicative control is distributed across the encounter. The present article addresses a related but different issue. Epistemic authority concerns whose knowledge claims and account of the clinical situation are treated as credible and action-guiding, whereas affective recognition concerns whether communicatively available distress is noticed, acknowledged or explored. The two lenses may interact, but the present analysis examines affective recognition rather than the distribution of epistemic authority. A patient’s symptoms may be reported and acted upon while their psychological distress—expressed through tone, hesitation, repetition, indirect disclosure or culturally specific idioms—remains unnoticed. Accurate clinical-content relay and affective recognition are therefore distinct communicative achievements, and an interpreter-mediated encounter may succeed in one while failing in the other.

This distinction also has implications for healthcare equity. Language has increasingly been conceptualised as a social determinant of health because linguistic accessibility shapes patients’ ability to obtain information, participate in care and communicate clinically relevant experiences [12]. From the perspective of translation justice, meaningful access cannot be reduced to the formal availability of translated or interpreted information; it must also enable patients to exercise substantive rights, including equitable access to healthcare and the ability to make their needs visible within institutional encounters [13]. Although Larroyed develops this argument primarily in relation to crisis settings, its underlying principle is relevant here: linguistic mediation is not merely procedural but may determine whether important dimensions of a patient’s experience become available for recognition and response.

The distinction between informational transfer and emotional recognition points towards different forms of intervention. Professional interpreting and training in triadic communication remain essential for improving the accuracy, safety and inclusiveness of multilingual healthcare [6,7]. However, recognising distress requires additional communicative and organisational capacities: interpreters must be able to preserve clinically relevant affective meaning, healthcare professionals must be attentive to emotional cues, and non-mental-health departments must have appropriate pathways for further assessment or referral. Research on interpreter–provider collaboration has shown that communication is jointly managed and that interpreters and clinicians may differ in their understanding of their roles, expertise and control over the interaction [14]. Moreover, work specifically examining interpreters’ emotion-related activity suggests that emotional meaning forms part of healthcare interpreting rather than an incidental element outside professional practice [15].

Against this background, the present study re-analyses a purposive subset of consultations from the Intercomsalud corpus using a coding scheme developed specifically to examine psychological distress. It addresses two questions:

1. When patients or caregivers express psychological distress, grief, anxiety, fear or trauma-related concerns during interpreter-mediated hospital consultations, how are these expressions conveyed across languages?

2. When distress becomes communicatively available, how do healthcare professionals working outside specialist mental health services respond: do they acknowledge it, explore it, refer the patient for further support or allow the interaction to move on without further response?

By distinguishing the relay of biomedical content from the recognition of emotional needs, the study examines how interpreter-mediated communication may facilitate—or constrain—the early identification of psychological distress in routine multilingual hospital care.

## 2. Conceptual Framework

### 2.1. Mental Health Recognition Outside Psychiatric Care

A substantial body of research shows that psychological distress may be missed, minimised or attributed exclusively to physical illness when it presents in general healthcare rather than specialist mental health services [1,3,16]. Somatic presentations, time pressure, competing biomedical priorities and the absence of routine assessment can all contribute to under-recognition. These difficulties are particularly relevant for children, older adults, patients living with chronic illness or disability and individuals experiencing social vulnerability, whose psychological needs may be overshadowed by the condition that brought them into care [3].

Non-mental-health departments should therefore not be regarded as peripheral to mental health integration. For many patients, they constitute the principal or only point of contact with the health system and consequently offer important opportunities for early recognition, brief intervention and referral. This understanding is consistent with the World Health Organization’s emphasis on prevention, integrated services and care organised around people’s life-course needs rather than around isolated diseases or professional sectors [4,5]. The WHO framework further stresses that people-centred care requires responsive services, effective communication and coordination within and beyond individual clinical settings [5].

### 2.2. Language Barriers and Interpreter-Mediated Healthcare

For patients who do not share a language with healthcare professionals, access to care is mediated by the availability and quality of linguistic support. Language barriers have been associated with difficulties in comprehension, participation, diagnosis, informed consent and patient safety [6,7]. Professional interpreters can reduce some of these risks and improve communication and care quality when compared with no interpreter or ad hoc language assistance [6].

Research on interpreter-mediated healthcare has also demonstrated that interpreters do not function as neutral conduits through which pre-existing meanings pass unchanged. They participate in the coordination of turn-taking, the management of information, the clarification of meaning and the negotiation of authority [10,11,14]. Hsieh’s work on provider–interpreter collaboration shows that both parties may seek to manage the content and process of the consultation, making effective communication dependent on their ability to coordinate their communicative goals and professional roles [14].

Most healthcare-interpreting research has nevertheless concentrated on the transfer of clinical content, including symptoms, diagnoses, treatment instructions and informed consent [6,10,11]. Affective meaning—how patients experience and respond emotionally to illness, risk and treatment—has received less sustained attention as an independent communicative outcome. Psychological distress may be conveyed explicitly, but it may also be expressed through repetition, pauses, hesitation, lowered voice, changes in narrative organisation or culturally situated formulations. Such features cannot always be separated neatly from the propositional content of the utterance.

Hsieh and Nicodemus conceptualise interpreters’ engagement with emotion as a form of professional “emotion work”, highlighting that interpreters may need to recognise, manage and communicate emotional meaning within healthcare encounters [15]. Their account provides a particularly relevant foundation for the present study because it challenges the assumption that fidelity can be evaluated only at the level of factual or terminological correspondence.

### 2.3. Language Access, Health Equity and Translation Justice

Language access is increasingly understood as an issue of health equity rather than simply a technical support service. Flores identifies language barriers as a significant obstacle to healthcare access and quality, while Federici conceptualises language as a social determinant of health whose effects can compound existing social and economic inequalities [7,12].

From this perspective, the presence of an interpreter does not by itself guarantee equitable access. Formal access may be achieved when information is translated, while substantive access remains limited if patients cannot communicate concerns that affect clinical judgement, participate meaningfully in decisions or make emotional and psychosocial needs visible. Translation justice extends this argument by treating adequate linguistic mediation as a condition for the effective exercise of rights rather than as an optional procedural accommodation [13]. Larroyed’s analysis focuses on crises and human-rights protection, but its central premise—that inadequate access to translation may prevent individuals from exercising rights to information, equality and healthcare—offers a useful framework for multilingual clinical settings [13].

In interpreter-mediated healthcare, equitable access should therefore be assessed not only through the accurate communication of biomedical information but also through the patient’s opportunity to express concerns, fears and vulnerabilities that may be relevant to care. This does not imply that interpreters should diagnose psychological distress or assume the clinician’s role. It does, however, mean that clinically significant affective meaning should remain communicatively available to those responsible for assessment and response.

### 2.4. Professional and Non-Professional Interpreting

When professional interpreters are unavailable, family members, acquaintances or bilingual staff may mediate communication [9,17]. Existing research has identified concerns regarding accuracy, confidentiality, autonomy and role boundaries in ad hoc interpreting [6,7]. Emotionally sensitive information may present additional complexities because relatives are not detached participants: they may share the patient’s distress, seek to protect them or influence how difficult information is presented.

However, the present dataset does not contain a sufficient number of verified family-interpreted consultations to support a systematic comparison between professional and non-professional interpreting. Accordingly, interpreter status is treated here as an important contextual issue rather than as a variable tested in the current analysis. The study instead concentrates on whether distress-related meaning remains available during the consultation and on what occurs once it has been conveyed.

### 2.5. From Clinical-Content Relay to Affective Recognition

The framework developed for this study distinguishes two related but analytically separate dimensions of multilingual healthcare communication. The first is clinical-content relay, referring to the communication of symptoms, diagnoses, risks, treatment options and instructions. The second is affective recognition, referring to whether expressions of fear, grief, anxiety, hopelessness or psychological vulnerability become available to the healthcare professional and receive an observable response.

This distinction does not imply that biomedical and emotional communication are independent in practice. On the contrary, they often coexist within the same utterance. Its analytical value lies in allowing the study to examine consultations in which factual information is successfully communicated while the emotional significance attached to that information is reduced, overlooked or left unexplored.

The study therefore treats the recognition of psychological distress as a communicative outcome that should not be inferred automatically from informational accuracy. This approach links healthcare-interpreting research on interactional coordination [14] and emotion work [15] with broader frameworks of language access, translation justice and integrated people-centred care [5,12,13].

## 3. Materials and Methods

### 3.1. Corpus

This study draws on the Intercomsalud corpus of 38 audio- and video-recorded consultations collected at Hospital Universitario La Paz, Madrid, between January and June 2025, previously analysed for power positioning, interpreter agency, and epistemic authority in a companion study (see Introduction). The full corpus spans a range of hospital departments and services, the large majority non-psychiatric (paediatrics, obstetrics and gynaecology, oncology, nephrology, ophthalmology, infectious diseases, cardiology, nutrition, urology, and social work), alongside a small number of psychology consultations offered to families under acute stress; one of the latter is included here specifically as a contrast case (Section 4.3), since it allows the pattern documented in non-psychiatric departments to be set against an encounter where distress recognition was the explicit purpose of the visit. The language distribution across the full corpus is Arabic (n = 16), Chinese (n = 8), English (n = 7), Russian (n = 5), and Ukrainian (n = 2). The study received ethical approval from the Ethics Committees of the Universidad de Alcalá and Hospital Universitario La Paz. All participants provided written informed consent prior to recording. Data were anonymised in accordance with LOPDGDD 3/2018 and the EU General Data Protection Regulation.

The analysis reported here does not cover the full corpus. A consultation entered the data-collection process whenever a healthcare professional telephoned the project to request interpretation for a patient. Each recorded consultation involved one healthcare professional and one interpreter. Patients and interpreters had no prior relationship and were introduced immediately before the consultation. Research assistant Carmen Merino-Cabello accompanied the interpreter, explained the project orally, provided the ethics-approved Participant Information Sheet and consent form, arranged oral interpretation of the project information into the patient’s language, and collected written informed consent before recording. Neither she nor the other researchers had a prior relationship with the patients. Because recruitment was triggered only after a healthcare professional requested the service, no denominator of potentially eligible patients, refusals or dropouts was recorded. No patient contributed a repeat consultation. Consultation duration was determined by clinical need and was not fixed by the research protocol. The present study is an exploratory, hypothesis-generating analysis of fifteen consultations (Arabic n = 6, Chinese n = 2, English n = 5, Russian n = 1, Ukrainian n = 1) selected because verified transcripts were available at the time of analysis. Selection was therefore neither random nor exhaustive and may have introduced both service-request and transcript-readiness bias: inclusion depended first on a healthcare professional requesting the interpreting service and then on a verified transcript being available. The subset cannot be treated as representative or theoretically saturated. Additional transcripts and field notes were reviewed but excluded when transcription inconsistencies required checking against recordings or when no dialogue was available to code. The final set of fifteen consultations was determined after this screening of the available transcripts and field notes, rather than fixed before the excluded materials were reviewed. These exclusions may have introduced additional selection bias beyond transcript readiness by favouring encounters with more complete, internally consistent and readily interpretable documentation. The eight cases described narratively were selected to illustrate variation across the coding categories, including explicit distress, observable cues, ambiguous responses, full/partial/non-relay and differing observable provider responses; they were not selected to estimate prevalence or compare departments, languages or professionals.

Although these consultations derive from the same data-collection effort as a separate study examining power asymmetries and epistemic authority, the present analysis addresses a different research question and uses an independently developed coding framework. The unit of analysis in this study is the communication and clinical uptake of expressions of psychological distress.

### 3.2. Coding Scheme

A coding scheme was developed iteratively for this study. Distress evidence was first classified by evidential status: (a) explicit verbal distress (direct statements of fear, anxiety, grief, hopelessness or trauma-related concern); (b) observable emotional cues documented in the recording or contemporaneous observation notes (e.g., crying, shaking, marked hesitation or repeated concern); or (c) researcher-inferred distress, used only when an interpretation depended on context, tone or a culturally situated expression. Silence, brief replies, gaze or hesitation alone were not coded as distress without corroborating verbal or observational evidence; such cases were coded as absent/ambiguous. Relay was coded as full when both the substantive concern and its clinically relevant affective force were preserved; partial when the concern remained recognisable but clinically relevant intensity, repetition, qualification or affective meaning was materially reduced; and non-relay when the distress-related meaning did not become available to the healthcare professional. Minor lexical differences that did not change clinical or affective meaning did not trigger a partial-relay code. Provider response captured only what was observable in the recording: acknowledgement/exploration, reassurance, indicated further assessment or referral, or no observable response. The operational coding protocol is provided in Appendix A.

Consultations were transcribed manually, and the qualitative materials and codes were managed in MAXQDA 2026. Candidate distress-related excerpts were independently coded from translator-reviewed transcripts by Carmen Merino-Cabello, a PhD candidate in the Doctoral Programme in Modern Languages: Research in Linguistics, Literature, Culture and Translation at Universidad de Alcalá, and by a second reviewer; development of the coding framework was discussed with the corresponding author. The interpreter was the participant with competence in the patient’s language during each consultation; neither the authors nor the research assistant had competence in the patient languages analysed. After recording, translators competent in the respective languages reviewed the manually prepared transcripts. The three top-level domains—distress expression, relay fidelity and healthcare-professional response—were specified deductively from the research questions and conceptual framework. Their subcategories, thresholds and uncertainty rules were refined inductively through application to the consultation data. The two coders subsequently compared their classifications in a consensus-adjudication meeting. Thus, 100% of candidate distress-related excerpts underwent consensus review. Where initial classifications differed, discussion addressed evidential status, relay fidelity and observable response until consensus was reached; unresolved uncertainty was retained as absent/ambiguous rather than resolved by inference. Original recordings were revisited when tone, turn order, speaker attribution or relay classification could not be assessed from text alone. Formal inter-rater reliability was not calculated because this was an exploratory interpretive analysis. A separate prospective count of excerpts with discrepant initial classifications was not maintained; consequently, a reliable disagreement percentage cannot be reconstructed retrospectively.

### 3.3. Analytic Procedure

Coded instances were compiled case by case in MAXQDA 2026 rather than aggregated statistically. The analytic sequence was: identify the evidential status of a possible distress expression; compare the source and interpreted utterances in manually prepared transcripts subsequently reviewed by translators competent in the respective languages and, where needed, revisit the recording; classify relay as full, partial or absent; and describe only the healthcare professional response observable before the recording ended. The reviewed subset consisted of project-arranged interpreters who supplied the relevant patient-language competence during the encounters. Neither the authors nor the research assistant spoke the patient languages; their analysis therefore relied on the interpreter-mediated record and the subsequent translator review of the transcripts. Transcripts were not returned to patients for checking because the project did not retain their contact details; no participant checking of findings was undertaken for the same reason. Family-interpreted consultations were excluded because verified transcripts were unavailable. Uncertainty was recorded explicitly and did not support a positive finding. The COREQ (Consolidated Criteria for Reporting Qualitative Research) Checklist is provided as Supplementary Material.

## 4. Results

Fifteen consultations were reviewed in this exploratory, hypothesis-generating analysis: six in Arabic (two Obstetrics, two Nutrition, Urology and Psychology), five in English (Gynaecology, Oncology and Plastic Surgery), two in Chinese (Infectious Diseases and Paediatrics), one in Russian-medium interpreting for a Ukrainian-speaking patient (Oncology), and one in Ukrainian-medium interpreting (Paediatrics). Eight were selected for detailed narrative presentation because, together, they illustrated the range of coding outcomes; seven were not narrated because they contained no codeable distress or repeated an already illustrated absent/ambiguous minimal-response pattern. Table 1 reports the full case basis. The table is descriptive and should not be read as a frequency comparison.

### 4.1. Distress Expression and Relay Fidelity

In an oncology consultation conducted through Russian-medium interpreting, the patient raised pain as a concern on several occasions during the encounter. The interpreter conveyed the pain complaint to the provider once, without conveying the patient’s repeated return to it, a pattern consistent with the interpreter’s general tendency, noted independently in the observation record, to summarise rather than render utterances in full. When the patient separately mentioned having lost several dental fillings as a side effect of chemotherapy, the provider responded with a shrug and a joke, redirecting her to see a dentist. Only after the consultation ended did the patient tell the research team, not the clinical staff, that she had disliked the provider’s manner and had unasked questions about diet in relation to the fillings, having felt the topic had been brushed aside. The distress and the unmet informational need existed; neither was relayed, addressed, or, in the second case, even raised within the clinical encounter itself.

In a paediatric consultation conducted through Ukrainian-medium interpreting, a mother was observed to be visibly nervous throughout, speaking quietly and avoiding eye contact, while being asked to consent to a second vaccine dose for her adolescent child following a serious transplant complication. The provider’s delivery was louder and more insistent than the mother’s speech, despite the provider explicitly stating that she understood the mother’s fear. The interpreter’s rendering was tonally softer than the provider’s delivery, more measured, but no less insistent on the substantive point, meaning the emotional register of the exchange was altered in relay even though its persuasive intent was preserved. Later in the same consultation, the provider read aloud from English-language research abstracts to justify the vaccination recommendation; under the resulting time pressure, the interpreter’s relay was reduced to isolated key phrases (“a lot of risk”, “high mortality rate”), and the research observer, who had some independent knowledge of Ukrainian, judged the translated version potentially contradictory relative to the source. Here, distress was visible in the patient’s behaviour and implicitly acknowledged in the provider’s words, yet the interpreting process available in that moment could not preserve both the clinical detail and the emotional tenor of the exchange at once.

An Arabic-mediated obstetric consultation provides a suggestive sequence in which observable responses varied within one encounter. The patient directly stated fear on three occasions. One received minimal acknowledgement, one prompted explanation and reassurance, and one was not visibly taken up before the topic changed. Because speaker attribution in this transcript was inferred partly from turn order rather than a complete speaker key, the recording was revisited; residual uncertainty remains. The sequence is therefore treated as illustrative, not demonstrative evidence of a stable recognition pattern.

By contrast, a consultation in Chinese-medium interpreting concerning a referral for an orthopaedic device (an infectious diseases follow-up) showed no codeable distress expression at all: the exchange was procedural, turn-taking was respected, and contact information was relayed accurately and completely. This consultation illustrates that accurate relay of procedural information may occur in the absence of observable distress-related communication.

### 4.2. Cases of Effective Recognition and Acknowledgement

Not every non-psychiatric case in this subset showed the pattern documented above. Two cases showed a provider or interpreter actively working to recognise and address distress rather than let it pass.

One illustrative Arabic-medium obstetric consultation showed sustained acknowledgement. The patient stated that she was very afraid; the healthcare professional explained the risk and available precautions, returned explicitly to the fear, and closed with personalised reassurance. The interpreter rendered the fear and reassurance in full. Juxtaposition with the other obstetric case is a hypothesis-generating contrast only: two cases cannot establish a department effect or attribute variation to the healthcare professional, interpreter, patient or clinical context.

A second, smaller instance came from an English-medium plastic-surgery consultation, where the interpreter offered reassurance before accurately relaying the clinical discussion. Minimal replies in this and a related oncology consultation (e.g., ‘Ok’) were classified as absent/ambiguous, not as evidence that distress was present but unexplored. Transcript-level analysis cannot distinguish calm acceptance, prior processing or muted reaction; the cases therefore demonstrate a limit of the data rather than a recognition failure. Resolving this ambiguity would require post-consultation interviews with patients or caregivers in their preferred language, ideally combined with interviews with interpreters and healthcare professionals, patient-reported measures, clinical-record review and longitudinal follow-up.

### 4.3. A Contrast Case: Full Recognition in a Psychology Consultation

One consultation in this subset was itself a psychology appointment, offered to a mother separated from a seriously ill child during an extended immigration process. Unlike every non-psychiatric case above, distress here was not incidental to the visit but its explicit subject, and the difference in relay and response was stark. The patient’s distress was disclosed at length and in her own words (“I sleep but I don’t sleep, I eat but I don’t eat, I am always anxious, nervous, and shaking”), and the interpreter rendered this fully, without compression or softening. The provider not only acknowledged it but actively extended it, naming the toll on the patient’s other children as well (“we’re talking about this affecting not only you, but your children there too, even changing how they behave”), and returning to it across the encounter rather than moving past it once raised.

This psychology consultation is an uncontrolled, suggestive contrast. Its interpreter, healthcare professional, patient and clinical purpose all differ from the non-psychology cases, so it cannot establish that departmental purpose caused the observed pattern. It raises the possibility that a consultation explicitly organised around emotional assessment may facilitate sustained recognition, but alternative explanations—including individual communication skill and case-specific factors—remain plausible.

### 4.4. Provider Response Patterns

Across the non-psychiatric cases, responses observable within the recordings varied from sustained acknowledgement to minimal or no visible uptake. No referral was observed or mentioned before recording ended. This is not evidence that referrals did not occur later, in the medical record or through pathways outside the dataset; downstream clinical action was not measured. The small purposive sample cannot support comparisons by department, language or professional profile.

## 5. Discussion

### 5.1. Affective Recognition as a Distinct Dimension of Language Access

This exploratory study extends previous research by identifying a hypothesis for further testing: accurate clinical-content relay may coexist with variable observable recognition of distress. The present dataset primarily illustrates this one-directional pattern and does not include a verified inverse case—distorted biomedical content with preserved affective recognition—sufficient to demonstrate empirical independence of the two dimensions. The distinction should therefore be regarded as an analytically useful, tentative framework rather than a validated two-dimensional model.

Across the analysed encounters, symptoms, risks, procedures and treatment options were generally conveyed in recognisable form. Some explicit distress expressions and corroborated observable cues were not consistently explored; brief replies, gaze and hesitation without corroboration were treated as absent/ambiguous. These observations are qualitative and do not establish that emotional meaning was statistically more likely than factual content to be reduced.

This interpretation is consistent with Hsieh and Nicodemus’s account of emotion work in healthcare interpreting, which suggests that emotional meaning forms part of the interactional labour of interpreted encounters rather than an optional supplement to “real” clinical content [15]. It also complements Hsieh’s analysis of provider–interpreter collaboration by showing that the coordination of communicative goals has consequences not only for informational control but also for which aspects of the patient’s experience become clinically salient [14].

The psychology consultation aligns with the possibility that an explicit emotional-assessment purpose may facilitate affective recognition, but the contrast is uncontrolled and speculative. It shows only that sustained recognition was possible in this recorded encounter; it cannot attribute the difference to department or organisational purpose.

### 5.2. From Formal Language Access to Equitable Participation

The findings also invite a broader interpretation of language access. Providing an interpreter is essential, but formal access to linguistic mediation does not automatically produce equitable participation. Flores’s work on language barriers and Federici’s conceptualisation of language as a social determinant of health both demonstrate that linguistic conditions can shape access, safety and health outcomes [7,12].

The present analysis suggests that this principle also applies to psychological and emotional dimensions of care. A patient may receive information about diagnosis and treatment while remaining unable to communicate the personal significance of that information in a way that prompts a clinical response. From the perspective of translation justice, access is therefore substantive only when linguistic mediation enables patients to make clinically relevant needs visible and to participate meaningfully in the encounter [13].

This does not mean that every expression of fear or uncertainty should be treated as evidence of a mental disorder or result automatically in referral. Emotional responses may be proportionate to difficult diagnoses, invasive procedures or uncertain outcomes. The clinical issue is not whether distress is eliminated, but whether it is noticed sufficiently for the professional to decide whether reassurance, further questioning, psychosocial support or specialist assessment is appropriate.

### 5.3. Implications for Interpreter Education and Clinical Practice

The observations suggest hypotheses for interpreter education and hospital practice rather than established intervention effects. Training could address preservation of clinically relevant emotional meaning and transparent communication of ambiguity, while future studies should evaluate whether such approaches improve recognition or outcomes.

Such training should not ask interpreters to diagnose distress or independently determine clinical relevance. Instead, it should equip them to avoid unnecessary affective flattening and to communicate ambiguities transparently when emotional meaning cannot be reproduced through straightforward propositional equivalence. This proposal is consistent with scholarship describing healthcare interpreting as coordinated interaction and emotion work rather than mechanical linguistic substitution [14,15].

The variation observed suggests that future research should examine how healthcare-professional communication, interpreter practice, clinical urgency and encounter structure influence recognition. The present sample cannot identify causal factors or compare professional profiles.

Integrated, people-centred care may be supported by brief distress-recognition prompts, guidance for interpreted interaction and accessible options for further assessment when indicated. These are practice implications suggested by the findings, not effects demonstrated by this study.

No referral to psychology, social work or equivalent support was observed within the recorded non-mental-health consultations. The study did not include chart review or follow-up and therefore cannot determine whether later referrals or other support occurred. Downstream referral behaviour is a significant evidence gap and is not treated as an outcome of this analysis.

### 5.4. Strengths, Limitations and Future Research

A principal strength of this study is its analysis of naturally occurring interpreter-mediated consultations recorded in routine hospital care. The corpus includes several clinical departments and language combinations, allowing the study to examine psychological distress as it emerged within consultations not primarily designed to assess mental health.

Several limitations must be acknowledged. First, this is a service-request and transcript-readiness sample from one hospital: cases entered data collection when a healthcare professional requested interpretation, and the analysed subset required a verified transcript. It cannot establish prevalence or comparisons across departments, languages, interpreters or healthcare professionals. Second, the case contrasts are illustrative and hypothesis-generating, not controlled comparisons. Third, coding required interpretive judgement, and no formal reliability statistic was calculated. Fourth, the authors and research assistant did not speak the patient languages; cross-language analysis relied on the interpreter-mediated record and subsequent review of transcripts by translators competent in the respective languages. Residual uncertainty was retained, and the Ukrainian paediatric and first caesarean cases warrant particular caution. Fifth, transcript and recording data capture only observable interaction and cannot establish unexpressed recognition, documentation, later referral or follow-up. Sixth, Arabic and English consultations predominate, and the subset is too small and uneven to identify language-specific patterns or culturally specific idioms reliably.

Second, the subset was selected according to the availability of verified transcripts. Consultations requiring further checking and cases documented only through field notes were excluded. The sample therefore reflects transcript readiness rather than theoretical saturation or representativeness.

Third, coding was conducted during an exploratory phase. All candidate distress-related excerpts were independently coded by two reviewers and subsequently underwent consensus adjudication; however, a separate count of discrepant initial classifications and formal inter-rater reliability statistics were not recorded. This limits the quantitative assessment of category stability.

Fourth, some judgements concerning tone, hesitation and visible distress necessarily involve interpretation. This is especially important where speaker roles were not consistently labelled or where the researcher did not have full proficiency in the relevant language. This is particularly relevant to the first caesarean case discussed in Section 4.1, in which speaker attribution was inferred from turn order rather than an explicit speaker key; potentially ambiguous speaker attributions were checked against the original recordings before final analysis.

Fifth, the study captures only distress that became observable in recordings or field notes. It cannot identify emotions that patients withheld or that were expressed outside the recorded consultation. Future research should combine interactional analysis with post-consultation patient or caregiver interviews conducted in the participant’s preferred language, interviews with interpreters and healthcare professionals, patient-reported measures, clinical-record review and longitudinal follow-up.

Finally, the present subset did not permit comparison between professional and non-professional interpreters. Future analysis of the full Intercomsalud corpus should examine whether interpreter status, clinical department, consultation length and the presence of explicit distress-recognition procedures influence affective relay and professional response.

## 6. Conclusions

This exploratory study identifies a hypothesis-generating distinction between clinical-content relay and observable affective recognition. In fifteen purposively selected consultations, biomedical and procedural information was generally conveyed in recognisable form, while the treatment of explicit distress and corroborated emotional cues varied. The sample cannot establish comparative frequencies, causal mechanisms or institutional patterns.

Some consultations showed sustained acknowledgement across languages, whereas others showed minimal or no observable uptake. This practice variation suggests possible inconsistency worthy of systematic study; it does not demonstrate a system-wide absence of recognition practices. The psychology case is a suggestive contrast, not evidence of a department effect.

Effective language access should consequently be understood as more than the accurate transmission of clinical information. In line with approaches that treat language as a social determinant of health and translation as a condition of substantive access, equitable multilingual care requires patients to be able to communicate emotional and psychosocial concerns that may influence clinical judgement [12,13].

The tentative distinction between clinical-content relay and affective recognition suggests questions for interpreter education, healthcare-professional training and service organisation. Proposed prompts, training and assessment pathways should be evaluated prospectively before protective effects are claimed.

Future research should test the coding framework in larger, independently coded and linguistically balanced samples, deliberately search for inverse patterns between clinical and affective relay, and combine recordings with post-consultation interviews involving patients or caregivers, interpreters and healthcare professionals, together with patient-reported measures, chart review and longitudinal follow-up, to resolve ambiguous minimal responses and assess recognition and downstream action.

## Figures and Tables

**Table 1:** Descriptive matrix of all fifteen consultations in the analysed subset. For the seven non-narrated cases, the retained analytic summary did not preserve every case-level category separately; these fields are reported conservatively as not individually recoverable rather than reconstructed retrospectively.

Observable Response	Relay	Distress Evidence	Interpreter	Language	Department	Case
Limited observable uptake	Partial/non-relay	Explicit repeated pain; post-visit dissatisfaction	Project-arranged	Russian for Ukrainian patient	Oncology	C01
Persuasive response; no further observable assessment	Uncertain/partial tonal relay	Observable cues; provider named fear	Project-arranged	Ukrainian	Paediatrics	C02
Mixed: minimal, substantive, then no observable uptake	Not individually recoverable from the retained analytic summary	Explicit fear repeated three times	Project-arranged	Arabic	Obstetrics	C03
Not applicable	Not applicable	No codeable distress	Project-arranged	Chinese	Infectious Diseases	C04
Sustained acknowledgement/reassurance	Full relay	Explicit fear	Project-arranged	Arabic	Obstetrics	C05
No distress response required/observable	Not applicable; interpreter reassurance observed	Absent/ambiguous minimal response	Project-arranged	English	Plastic Surgery	C06
No distress response required/observable	Not applicable	Absent/ambiguous minimal response	Project-arranged	English	Oncology	C07
Sustained exploration (contrast case)	Full relay	Explicit distress	Project-arranged	Arabic	Psychology	C08
No distress-specific response documented in the retained summary	Not applicable or not individually recoverable	No codeable distress or absent/ambiguous minimal response; individual classification not retained	Project-arranged	Arabic	Nutrition	C09
No distress-specific response documented in the retained summary	Not applicable or not individually recoverable	No codeable distress or absent/ambiguous minimal response; individual classification not retained	Project-arranged	Arabic	Nutrition	C10
No distress-specific response documented in the retained summary	Not applicable or not individually recoverable	No codeable distress or absent/ambiguous minimal response; individual classification not retained	Project-arranged	Arabic	Urology	C11
No distress-specific response documented in the retained summary	Not applicable or not individually recoverable	No codeable distress or absent/ambiguous minimal response; individual classification not retained	Project-arranged	Chinese	Paediatrics	C12
No distress-specific response documented in the retained summary	Not applicable or not individually recoverable	No codeable distress or absent/ambiguous minimal response; individual classification not retained	Project-arranged	English	Gynaecology, Oncology or Plastic Surgery; individual mapping not retained	C13
No distress-specific response documented in the retained summary	Not applicable or not individually recoverable	No codeable distress or absent/ambiguous minimal response; individual classification not retained	Project-arranged	English	Gynaecology, Oncology or Plastic Surgery; individual mapping not retained	C14
No distress-specific response documented in the retained summary	Not applicable or not individually recoverable	No codeable distress or absent/ambiguous minimal response; individual classification not retained	Project-arranged	English	Gynaecology, Oncology or Plastic Surgery; individual mapping not retained	C15

## Data Availability

The datasets generated and analysed during the current study are not publicly available because they contain identifiable clinical interactions and are protected under the informed consent agreements and applicable data protection legislation (LOPDGDD 3/2018 and the General Data Protection Regulation). De-identified excerpts supporting the findings of this study may be available from the corresponding author upon reasonable request, subject to ethical approval and institutional requirements.

## Supplementary Materials

The following supporting information can be downloaded at: https://www.mdpi.com/article/10.3390/healthcare14182945/s1, File COREQ (Consolidated Criteria for Reporting Qualitative Research) Checklist.

## Appendix A. Operational Coding Protocol

Step 1—Evidence status. Code explicit verbal distress only when the speaker directly names fear, anxiety, grief, hopelessness or trauma-related concern. Code an observable cue only when documented in audio/video or contemporaneous notes and clinically relevant in context. Code researcher-inferred distress separately; do not convert silence, gaze, short replies or hesitation alone into a positive distress finding.

Step 2—Relay fidelity. Full relay preserves the concern and its clinically relevant affective force. Partial relay preserves a recognisable concern but materially reduces intensity, repetition, qualification or affective meaning. Non-relay means the distress-related meaning does not become available to the healthcare professional. Minor lexical variation without a meaningful clinical or affective change remains full relay.

Step 3—Observable response. Describe acknowledgement/exploration, reassurance, indicated further assessment/referral, or no observable response. Do not infer private recognition, documentation or action after recording.

Step 4—Uncertainty and adjudication. Revisit recordings for tone, speaker attribution or cross-language uncertainty. Discuss borderline cases and retain absent/ambiguous or uncertain labels when evidence is insufficient. Worked borderline example: a single-word ‘Ok’ without corroborating cues is absent/ambiguous, not unrecognised distress.
