# Supplementary material for: When Distress Goes Unseen: Mental Health Recognition Failures in Language-Discordant Consultations
Source: Healthcare (Basel). 2026 Sep 10;14(18):2945. doi: 10.3390/healthcare14182945 (PMC13606488; doi:10.3390/healthcare14182945)
Supplement: Supplementary file 1 [file healthcare-14-02945-s001.zip › healthcare-4489572-supplementary.pdf]

COREQ (Consolidated Criteria for Reporting Qualitative Research) Checklist

Manuscript: When Distress Goes Unseen: Mental Health Recognition Failures in Language-Discordant Consultations

Completion status: All items applicable to this analysis of naturally occurring clinical consultations have been completed. “Not applicable” is used where COREQ’s interview/focus-group wording does not correspond to the study design.

No.	Item	Guide question	Reported information / manuscript location
**Domain 1: Research team and reflexivity**			
1	**Interviewer/facilitator**	Which author/s conducted the interview or focus group?	Not applicable. The study analysed naturally occurring hospital consultations; the research team did not conduct research interviews or focus groups. Clinical encounters were conducted by the attending healthcare professionals and mediated by project-arranged interpreters. Methods 3.1 and 3.3.
2	**Credentials**	What were the researcher’s credentials?	The initial coder, Carmen Merino-Cabello, was a PhD candidate in the Doctoral Programme in Modern Languages: Research in Linguistics, Literature, Culture and Translation at Universidad de Alcalá. Carmen Pena-Díaz is affiliated with the FITISPos Research Group, Department of Modern Languages, Universidad de Alcalá; Fe Amalia García Santiago is affiliated with IdiPAZ, Hospital Universitario La Paz. Title page; Methods 3.2; Author Contributions.
3	**Occupation**	What was their occupation at the time of the study?	Carmen Merino-Cabello was a doctoral researcher/research assistant at Universidad de Alcalá. The authors’ institutional affiliations and study roles are reported on the title page and in Author Contributions.
4	**Gender**	Was the researcher male or female?	The primary coder/research assistant, Carmen Merino-Cabello, was a woman.
5	**Experience and training**	What experience or training did the researcher have?	Carmen Merino-Cabello was undertaking doctoral training in modern languages, including research in linguistics, literature, culture and translation. She performed initial coding in consultation with the corresponding author; a second author reviewed all candidate distress excerpts. The authors and research assistant did not speak the patient languages. Interpreters held the relevant competence during consultations, and translators competent in the respective languages subsequently reviewed the transcripts. Methods 3.2–3.3.
6	**Relationship established**	Was a relationship established prior to study commencement?	No researcher had a prior relationship with the patients. Patients and interpreters also did not know one another before the consultation and were introduced immediately beforehand. Written informed consent was obtained before recording. Methods 3.1; Informed Consent Statement.
7	**Participant knowledge of the interviewer**	What did participants know about the researcher?	Before the consultation, Carmen Merino-Cabello explained the project orally and provided the ethics-approved Participant Information Sheet and Informed Consent Form. The project explanation was interpreted into the participant’s language before written consent was collected. Methods 3.1; Informed Consent Statement.
8	**Interviewer characteristics**	What characteristics were reported about the interviewer/facilitator?	Not applicable to interviewing: researchers did not facilitate the clinical encounters. Reflexive characteristics relevant to coding—including training, disciplinary background and language competence—must be completed under Items 2–5. Methods 3.2–3.3.
**Domain 2: Study design**			
9	**Methodological orientation and theory**	What methodological orientation was stated to underpin the study?	Exploratory, hypothesis-generating qualitative re-analysis of naturally occurring interpreter-mediated consultations, using a purpose-built coding framework informed by interpreter-mediated interaction, emotion work, language access, translation justice and integrated people-centred care. Introduction; Conceptual Framework 2.1–2.5; Methods 3.2–3.3.

No.	Item	Guide question	Reported information / manuscript location
10	**Sampling**	How were participants selected?	Purposive transcript-readiness sampling: fifteen consultations were selected from a corpus of 38 because verified transcripts were available. Selection was neither random nor exhaustive and may introduce readiness bias. Methods 3.1; Strengths and Limitations 5.4.
11	**Method of approach**	How were participants approached?	A consultation entered the project whenever a healthcare professional telephoned to request interpretation for a patient. Carmen Merino-Cabello accompanied the interpreter, met participants face-to-face before the consultation, distributed the ethics-approved participant information and consent forms, and collected written informed consent before recording. Methods 3.1; Informed Consent Statement.
12	**Sample size**	How many participants were in the study?	Fifteen consultations were analysed. Each involved one healthcare professional and one interpreter, with no repeat patient consultations. The consultation-level language distribution was Arabic n=6, Chinese n=2, English n=5, Russian n=1 and Ukrainian n=1. Abstract; Methods 3.1; Table 1.
13	**Non-participation**	How many people refused or dropped out? Reasons?	Not available. The team was contacted only when a healthcare professional requested interpretation, so no denominator of potentially eligible patients, refusals or dropouts was recorded. Transcript exclusions are reported separately. Methods 3.1.
14	**Setting of data collection**	Where was the data collected?	Routine hospital consultations at Hospital Universitario La Paz, Madrid, Spain, January–June 2025, across several departments and services. Methods 3.1.
15	**Presence of non-participants**	Was anyone else present besides participants and researchers?	Each consultation involved one healthcare professional and one project-arranged interpreter, in addition to the patient/caregiver as applicable. Carmen Merino-Cabello accompanied the interpreter to the consultation and administered the information and consent process before recording. No additional healthcare professionals or interpreters participated. Methods 3.1.
16	**Description of sample**	What are the important characteristics of the sample?	Fifteen multilingual hospital consultations across Arabic, Chinese, English, Russian and Ukrainian, spanning Obstetrics, Nutrition, Urology, Psychology, Gynaecology, Oncology, Plastic Surgery, Infectious Diseases and Paediatrics. The subset used project-arranged interpreters; family-interpreted cases were excluded because verified transcripts were unavailable. Methods 3.1 and 3.3; Table 1.
17	**Interview guide**	Were questions, prompts or guides provided by the authors? Was it pilot tested?	Not applicable. These were naturally occurring clinical consultations, not researcher-led interviews; no research interview guide or prompts were administered. The analytic coding protocol is provided in Methods 3.2 and Appendix A.
18	**Repeat interviews**	Were repeat interviews carried out?	No. No patient contributed a repeat recorded consultation. These were clinical encounters rather than research interviews.
19	**Audio/visual recording**	Did the research use audio or visual recording?	Yes. The Intercomsalud corpus contains audio- and video-recorded consultations. Original recordings were revisited when tone, turn order, speaker attribution or relay classification could not be assessed from transcripts alone. Methods 3.1–3.3.
20	**Field notes**	Were field notes made during and/or after data collection?	Yes. Contemporaneous observation records/field notes were available for some cases and were used to contextualise observable conduct and transcript quality. A field-note-only bereavement case was excluded because it contained no recorded dialogue to code. Methods 3.1; Results 4.1.
21	**Duration**	What was the duration of the interviews or focus groups?	Consultations had no fixed research-defined duration; their length was determined by clinical need. These were clinical consultations rather than interviews.
22	**Data saturation**	Was data saturation discussed?	The sample was not selected for theoretical saturation. It reflects transcript readiness and supports illustration/hypothesis generation rather than prevalence estimates. Methods 3.1; Strengths and Limitations 5.4.

No.	Item	Guide question	Reported information / manuscript location
**23**	**Transcripts returned**	Were transcripts returned to participants for comment/correction?	No. Transcripts were not returned because the project did not retain participant contact details. Transcripts were reviewed by translators competent in the respective languages, and uncertain cases were checked against original recordings. Methods 3.1–3.3.
**Domain 3: Analysis and findings**			
**24**	**Number of data coders**	How many data coders coded the data?	Carmen Merino-Cabello conducted initial coding in consultation with the corresponding author. All candidate distress excerpts were reviewed by a second author, and differences were resolved through discussion. Formal inter-rater reliability was not calculated. Methods 3.2; Limitations 5.4.
**25**	**Description of the coding tree**	Did authors provide a description of the coding tree?	Yes. The coding framework covers: evidential status of distress (explicit verbal, observable cue, researcher-inferred/ambiguous); relay fidelity (full, partial, non-relay); and observable healthcare-professional response. Operational rules and a borderline example appear in Appendix A; Table 1 applies the categories.
**26**	**Derivation of themes**	Were themes identified in advance or derived from data?	A hybrid deductive–inductive approach was used. The three top-level domains—distress expression, relay fidelity and healthcare-professional response—were derived deductively from the research questions and conceptual framework. Subcategories, thresholds and uncertainty rules were refined inductively through application to the consultation data. Methods 3.2; Appendix A.
**27**	**Software**	What software, if applicable, was used to manage the data?	Consultations were transcribed manually. MAXQDA was used to manage the qualitative materials and coding. Methods 3.2–3.3.
**28**	**Participant checking**	Did participants provide feedback on the findings?	No. Participant checking was not undertaken because the project did not retain participant contact details.
**29**	**Quotations presented**	Were participant quotations presented and identified?	Yes. De-identified excerpts illustrate explicit fear, anxiety, reassurance and minimal responses. Cases are identified contextually by department/language rather than personal identifiers. Results 4.1–4.3.
**30**	**Data and findings consistent**	Was there consistency between data presented and findings?	The revised manuscript links each interpretation to case material, distinguishes explicit/corroborated evidence from absent/ambiguous responses, provides all-case context in Table 1, and confines claims to observable recorded interaction. Results 4.1–4.4; Discussion 5.1; Appendix A.
**31**	**Clarity of major themes**	Were major themes clearly presented?	Yes. Major findings are organised around distress expression and relay fidelity, effective recognition/acknowledgement, the uncontrolled psychology contrast, and observable response patterns. Results 4.1–4.4.
**32**	**Clarity of minor themes/diverse cases**	Were diverse cases or minor themes discussed?	Yes. The manuscript reports effective-recognition cases, a procedural case with no codeable distress, absent/ambiguous minimal-response cases, uncertainty in speaker attribution/translation, and an uncontrolled psychology contrast. Negative and ambiguous cases are used to bound rather than strengthen claims. Results 4.1–4.4; Limitations 5.4.
